# Evaluation of Oyster Mushroom (*Pleurotus ostreatus*) Production Using Water Hyacinth (*Eichhornia crassipes*) Biomass Supplemented with Agricultural Wastes

**DOI:** 10.1155/2022/9289043

**Published:** 2022-03-15

**Authors:** Nigistie Ejigu, Baye Sitotaw, Solomon Girmay, Hirut Assaye

**Affiliations:** ^1^Bahir Dar University, Department of Biology, Ethiopia; ^2^Bahir Dar University, Department of Applied Human Nutrition, Ethiopia

## Abstract

The cost of substrates has been one of the challenges for mushroom cultivation. The commonly used substrates for mushroom production are usually expensive. Substrates with a high biomass return that can pose environmental problems can be good alternatives for mushroom cultivation due to multiple advantages. In this regard, the potential use of water hyacinth biomass (a troublesome aquatic weed) as an alternative substrate is worthy of being studied. This study was aimed at evaluating the potential use of water hyacinth biomass for the production of oyster mushroom. The experiment was done in a completely randomized design with nine treatments and four replications. Water hyacinth biomass was supplemented with straw (wheat, *Triticum aestivum*, and *teff* or *Eragrostis Teff*) at a ratio of 1 : 1, 1 : 3, or 3 : 1. The developmental parameters including days elapsed for mycelium invasion (MI), pinhead formation (PF), and the first flush (FH) were monitored. Growth parameters (cap diameter (CD) and stalk length (SL)), a yield parameter (total weight of mushroom yield), and biological efficiency (BF %) were also recorded. Finally, the economic return (ER) of all the treatments was calculated. A one-way analysis of variance (ANOVA) was used to test the significance of variation between the different parameters on the production parameters. Means were separated using the Tukey test, when F-test from ANOVA was significant at *p* ≤ 0.05. It was observed that water hyacinth biomass alone or supplemented with wheat or *teff* straw provided promising performance on oyster mushroom development, growth, yield, and biological efficiency compared to the costly substrates (wheat and *teff* straw). Thus, water hyacinth can be considered as a low-cost substrate for mushroom cultivation and a means to control this aquatic weed from rapid spreading.

## 1. Introduction

Oyster mushroom (*Pleurotus ostreatus*) is one of the most common types of cultivated mushrooms in the world. It is the second largest commercially produced mushroom next to *Agaricus bisporus* globally, especially in Southeast Asia, India, Europe, and Africa [1–3]. Oyster mushroom cultivation has several advantages over other edible mushrooms. It grows fast under a wide range of temperature (10-30°C) and pH (6-8) [4]; secretes a wide range of enzymes that are capable of degrading lignocellulosic biomass of substrates [5]; demands a few environmental control; does not need composting of its substrate; can colonize substrates in a short period of time; and has high yield potential and high nutritional and medicinal values [6–9]. Moreover, the substrate used for its cultivation needs only pasteurization (does not require a more expensive method—sterilization), their fruiting bodies are not often attacked by diseases and pests, and generally, they can be cultivated in simple and cheap ways.

Biomass rich in complex carbohydrates such as cellulose, hemicelluloses, and lignins are the best substrates for mushroom cultivation [10]. Oyster mushrooms are commonly wood and other lignocellulosic decaying fungi [11]. They can also grow best on different lignocellulosic agricultural wastes such as wheat straw, *teff* straw, paddy straw, cotton waste, coffee pulp, and sugarcane bagasse [12]. However, all the aforementioned substrates are in demand (can also be used for other purposes) and relatively costly. The costs of substrates to grow mushrooms have thus been some of the challenges in the industry.

Several studies have been conducted to find out the best substrate (substrate composition) for oyster mushroom cultivation in terms of productivity, nutritional quality, and economic return [13–16]. Few studies highlight the advantage of using water hyacinth (*Eichhornia crassipes*) biomass as a substrate for mushroom production [17–19]. Murugesan et al. [17] demonstrated a promising yield of oyster mushrooms on water hyacinth biomass, owing to its ideal C: N ratio and low lignin content of its biomass.

Water hyacinth (*Eichhornia crassipes*) is a free-floating aquatic weed, which is characterized by rapid growth rates, large vegetative reproduction output, and a broad environmental tolerance [20]. It is a troublesome aquatic weed, threatening mainly freshwater lakes, such as Lake Tana (Ethiopia) exposed to aggravating factors. This weed has been managed via different methods such as chemical, physical, and biological methods [17]. One of the biological methods is using such biomass as a substrate for mushroom cultivation [21], and such “controlling by utilization approach” has recently gained great attention [22]. This approach can be very cheap and also help eradicate such aquatic weed through converting it into a value-added bioproduct. Accordingly, several attempts have been made to find out the possibility of using water hyacinth biomass as an alternative substrate for mushroom cultivation [3, 23–25]. At least, the water hyacinth biomass could feasibly be used to supplement other substrates [26]. While some efforts have been made to evaluate the potential use of water hyacinth biomass as an alternative substrate for oyster mushroom cultivation in other countries, this aspect has not been evaluated against *teff* straw (commonly available agricultural waste in Ethiopia).

Water hyacinth has been recognized as the most damaging aquatic weed in Ethiopia since 1965 [27]. In Lake Tana, its presence has been reported since 2011 [28]. Since then, water hyacinth has been adversely affecting the Lake Tana ecosystem. About 34,500 ha (15% of the Northern shore of Lake Tana) has been covered with the infestation of water hyacinth [29]. Several approaches including mechanical, biological, and chemical methods have been employed to control this weed. This study was aimed at evaluating the potential use of water hyacinth biomass for the production of oyster mushroom with the view of the multiple advantages that can be gained. Two types of commonly available cereal straw in the study area, namely, wheat (*Triticum aestivum*) and *teff* (*Eragrostis Teff*) straw were used as supplements and controls. Utilization of water hyacinth, collected from Lake Tana, for mushroom cultivation will have manifold advantages as the lake is at a high risk of deterioration due to the alarming expansion of this weed in the lake.

## 2. Materials and Methods

### 2.1. Description of the Study Area

This study was conducted in Bahir Dar city (the capital city of the Amhara region), located 565 km away from of Addis Ababa (the capital city of Ethiopia). This city is situated at the southern shore of Lake Tana, the biggest lake in Ethiopia. Bahir Dar is located at an altitude and longitude of 11°35′N and 37°23′E with a mean elevation of 1,800 meters above sea level. The average annual temperature and rainfall in the area are 19.6°C and about 1800 mm, respectively. Moreover, the average annual humidity of the area is 58%.

### 2.2. Treatments and Study Design

The experiment was conducted in a completely randomized design with nine treatments (Table 1). The study was conducted from January to June 2021 to evaluate the potential use of hyacinth biomass for the production of the oyster mushroom. Two agricultural wastes, namely, wheat and *teff* straw were used as controls and supplements (Table 1). Four replications with a total of 36 bags were used for the study.

### 2.3. Source and Handling of the Spawn

The spawn of *Pleurotus ostreatus* was obtained from a small enterprise working on spawn production and distribution in Addis Ababa and was carefully transported to Bahir Dar University, Department of Biology, microbiology laboratory. Upon arrival, the spawn was stored in a dark place at room temperature until inoculated into the different substrates.

### 2.4. Processing of the Substrates

The fresh water hyacinth was collected from Lake Tana, Ethiopia. The straw (wheat and *teff*) was collected from local farmers in the vicinity of Bahir Dar city. The root part of the water hyacinth was excluded and the remaining part was washed followed by drying it in open air [30]. Then, both the water hyacinth and the straw were chopped into small pieces (about 4-6 cm) and heat treated by immersing them into boiling water for 30 minutes, followed by drying in open air [30, 31].

Each treatment containing 1 kg substrate was prepared as indicated in Table 1. Before mixing the substrates, they were immersed separately into clean tap water and left overnight with the addition of 3% of CaCO_3_ [32]. After excess water was drained, they were dried in a shade until the required moisture content was achieved. The moisture content was checked through the squeezing of each substrate in the palm until no drop of water was observed [33]. Following this, single and mixed substrates were filled into 36 heat-resistant polypropylene bags with a size of 40x30 cm and autoclaved at 121°C for 30 minutes followed by cooling at room temperature for 12 hours [34].

### 2.5. Substrate Inoculation, Cultivation System, and Harvesting

After cooling, each bag was inoculated with 80 g of oyster mushroom seeds (*Pleurotus ostreatus)* [14]. The top opening of each bag was plugged with cotton and a tube was attached to it for adequate aeration. Then, all the bags were placed in a dark spawn running room at a temperature of 25-30°C and relative humidity of 70-80% [6]. The bags were supported by steel racks, which were disinfected with 70% alcohol.

After all the bags were fully colonized by mycelia, they were moved from the dark room to a room with light for fructification. Proper ventilation of the growth room was assured by opening the door and windows 2-3 times a day and using an air conditioner [6]. In this fruiting room, the temperature was thus lowered to 20-25°C. The floor of the fruiting room, mycelium containing substrates, and hanging materials were sprayed with water 3-4 times a day to maintain a high humidity level of 80-90% and to lower the temperature. In addition, 5-6 water containing baths were placed at each corner of the fruiting room [35].

Harvesting was performed when the fruiting bodies were well developed and the caps were fully opened when they were upright and curled [36]. This was performed by gently pulling or twisting the stalk from the substrate using a knife. Mushroom flushes were harvested three times during the total cropping period.

### 2.6. Data Collection and Analysis

Eight parameters were measured throughout the production process from the 36 bags. The developmental parameters including the time (days) elapsed for mycelium invasion (MI), pinhead formation (PF), and the first harvest (FH) were monitored. Growth (cap diameter (CD) and stalk length (SL)), yield (total weight of mushroom), and biological efficiency (BF %) were also recorded. Finally, the economic return (ER) on all treatments was calculated. From each bag, 50% of the fruiting bodies (flushes) were selected for the measurement of stalk length and cap diameter. Biological efficiency (BE) was determined as a ratio of the biological yield harvested to the dry weight of each substrate (1000 g) using the formula [37]:

(1) $$\begin{array}{c} \%BE=\frac{\text{Total weight of fresh mushroom}}{\text{Dry weight of substrate}}\times 100. \end{array}$$

The economic return was determined in terms of the Benefit-Cost (B: C) ratio [38] by considering only the total weight of mushrooms produced on each treatment. The test parameters (MI, PF, FH, CD, SL, and BE) and other considerations were omitted to calculate B:C ratio for the sake of simplification.

The data were analyzed using the SPSS version 26 software. A one-way analysis of variance (ANOVA) was used to test the significance of variation between developmental, growth, and yield parameters of oyster mushrooms in the different treatments. Means were separated using the Tukey test, when F-test from ANOVA was significant at *p* ≤ 0.05. The results were recorded as mean ± standard deviation and presented in tables.

### 2.7. Limitations of the Study

We are aware that this study is not free from limitations. Nitrogen content, C/N ratio, and carbohydrate content (cellulose, hemicellulose, lignin, etc.) of each substrate, as well as the number of fruiting bodies, dry matter content, protein content, etc. of the oyster mushroom produced, should have also been evaluated.

## 3. Results

### 3.1. Effects of Substrate Combinations on Developmental Parameters of Oyster Mushroom

The average elapsed days required to complete mycelium running (MI) in the spawned bags ranged from 21.50 to 31.25 days, with statistically significant differences across the treatments (*p* ≤ 0.05) (Table 2). The lowest MI was 21.50 days which was recorded in T1 (100% wheat straw). This was significantly different from all other treatments except T2 (100% *teff* straw) and T4 (75% wheat straw+25% water hyacinth). The maximum number of days for the completion of mycelium running was 31.25, which was recorded in T3 (100% water hyacinth), and was statistically different from T1, T2, T4, and T5 (50% wheat straw+50% water hyacinth) but was not significantly different from the remaining treatments.

The time (days) required to start pinhead formation (PF) of oyster mushrooms was significantly different (*p* ≤ 0.05) between the different substrates (Table 2). The longest PF was observed in T3 (6.25 days), but it was statistically different only with T2. The shortest time was observed in T2 (3.5 days), which was significantly different from all treatments except for T7, T8, and T9.

Similarly, the different substrates had significantly different (*p* ≤ 0.05) number of days taken until starting the first harvest (FH) after the opening of the bags (Table 2). The longest FH was recorded in T3 (10.25 days), though statistically different only in T2. The shortest FH (7.5 days) was observed in T2, which was statistically different from all the treatments except T7, T8, and T9.

### 3.2. Effects of Substrate Composition on Growth Parameters of Oyster Mushroom

Mean cap diameter (CD) and mean stalk length (SL) of oyster mushrooms were significantly different (*p* ≤ 0.05) between the treatments evaluated (Table 3). The highest mean CD (7.14 cm) was recorded in T1, which was significantly different from all the treatments except for T4 and T5. The lowest mean CD was recorded in T2 (1.65 cm), which was significantly different from all the treatments except for T3, T7, T8, and T9. Similarly, the mean longest stalk (3.69 cm) was recorded in T1 followed by T4 (3.41 cm), and the shortest mean stalk (1.29 cm) was recorded for T2. Generally, mean CD and SL showed a decreasing trend from the first to third flushes.

### 3.3. Effects of Substrate Composition on Yield and Biological Efficiency of Oyster Mushroom

The weight (yield) of the oyster mushroom in each treatment was recorded separately at the first, second, and third harvesting stages (flush), and the sum of the weight was considered as a total yield (Table 4). In all harvests, a declining trend of the yield was observed from the first to third harvesting stages.

The mean total yield (g) ranged from 350.09 ± 38.11 to 957.84 ± 25.14 with the top highest yields recorded in T1 (957.84 ± 25.14), T4 (830 ± 26.87), T5 (754 ± 33.90), and T6 (660.30 ± 32.97). There were significant differences in the yield between most treatments. The mean total yield recorded in T1, T4, T5, and T6 showed significant differences from the yield recorded in all other treatments (*p* ≤ 0.05). However, there were no significant differences in mean total yield between T3, T8, and T9; between T2 and T7; and between T7, T8, and T9.

As indicated in Table 4, the mean BE (%) ranged from 35.01 ± 0.01 to 95.78 ± 0.51. The highest meant total BE was obtained from T1 (100% wheat straw). It was significantly different from all other treatments in all harvesting stages. On the other hand, the lowest mean BE was observed in T2 (100% *teff* straw) and was significantly different from all other treatments except for T7, T8, and T9 (*p* ≤ 0.05). However, there were no statistically significant differences in BE between T3, T7, T8 and T9; between T4 and T5; and between T5 and T6.

### 3.4. Effects of Substrate Combinations on Economic Return from Each Treatment

As shown in Table 5, economic return was determined in terms of Benefit-Cost ratio (B: C) from each treatment. It is the ratio of gross income from the selling of mushrooms produced in each treatment to the total cost incurred to obtain and/or to process each substrate. A substrate with a higher value of B:C is taken as more preferable. In this study, the highest economic return (148) was obtained for 100% water hyacinth followed by the use of 25% *teff* straw+75% water hyacinth with a B: C value of 57.73. The lowest B:C ratio (15.77) was obtained for T2 (100% *teff* straw).

## 4. Discussion

Several studies have been conducted to select the best substrates or substrate combinations for oyster mushroom cultivation [3, 23–25]. Agricultural wastes such as wheat and *teff* straws are among the common substrates used for mushroom production. However, there has been a growing interest in the selection of substrates having multiple advantages. Accordingly, this study was aimed at evaluating the potential use of water hyacinth (*Eichhornia crassipes*), a troublesome aquatic weed, as an alternative substrate for the production of the oyster mushroom.

In this study, nine treatments (substrate compositions by weight) were evaluated based on selected developmental, growth, and yield parameters, as well as considering the economic return. The treatments included wheat straw, *teff* straw and water hyacinth biomass alone, and water hyacinth mixed with wheat and *teff* straws at a ratio of 1 : 3, 1 : 1, or 3 : 1 (Table 1). Wheat straw was selected for it is one of the commonly used substrates that give high mushroom yield, while *teff* straw was selected for this substrate is reported as the best substrate in terms of nutritional content and efficiency in pinhead formation.

### 4.1. Effects of Substrate Composition on Developmental Parameters

Generally, shorter periods of time elapsed for mycelial colonization, pinhead formation, and until the first flush are preferred outcomes. In addition, mycelial colonization is a prerequisite condition for fruiting since a successful growth of mycelium is a vital step in mushroom cultivation [39]. Based on the results of this study, significantly shorter periods of time (21 to 25 days) were elapsed for mycelium invasion (MI) in T1 (100% wheat straw) followed by T4 (75% wheat straw+25% water hyacinth), T2 (100% *teff* straw), and T5 (50% wheat straw+50%water hyacinth). On the other hand, longer periods of times (27 to 31 days) were elapsed for MI at substrates, which constitute water hyacinth, indicating that water hyacinth biomass may be challenging this developmental process. As a result, a pretreatment step may be required to make the biomass more available to the fungus [40].

Similarly, a varying length of periods of time (days) (3.50-6.25 days) were elapsed for pinhead formation in the different substrate compositions (PF). On average, shorter periods of time were elapsed for PF mostly in substrates containing *teff* straw. Similarly, Dubey et al. [41] demonstrated that the shortest time for pinhead formation was observed with *teff* straw (3 days). The substrates that have a lower C/N ratio and are rich in nitrogen, such as *teff* straw, are known to provide the fastest pinhead formation [41].

Regarding the time elapsed until harvesting the first flush (FH), varying periods of time (7.50-10.25 days) were observed between the different substrates as well. Similar to the results observed with the PF, substrates containing *teff* straw demonstrated a shorter period of time for FH, implying that *teff* straw may have some favoring attributes for these two developmental parameters. On the contrary, MI, PF, and FH took the longest times at 100% water hyacinth, suggesting that pretreatment of water hyacinth biomass may be required as explained earlier.

### 4.2. Effects of Substrate Composition on Growth Parameters

The two growth parameters measured, namely, cap diameter (CD) and stalk length (SL) of the oyster mushroom against the various substrate compositions (treatments) varied seemingly due to the different factors intrinsic to the substrate (wheat and *teff* straw and water hyacinth biomass). As far as biological efficiency (weight of biomass) is concerned, higher values for both parameters indicate better growth. Oyster mushrooms with larger caps and shorter stalks are considered better than those with smaller caps and longer stalks [42], which are other parameters, termed as economic efficiency. This is due to the fact that the stalks contained more insoluble dietary fibers than the caps. In addition, Kivaisi et al. [43] reported that the size of the cap is influenced by aeration and the amount of light, which were not controlled in this study. Larger CD and SL were obtained in the treatments containing wheat straw (Table 3). Water hyacinth biomass gave better CD and SL than *teff* straw, which was the expected outcome.

### 4.3. Effect of Substrate Composition on Yield and Biological Efficiency

Yield and biological efficiency (BE) are the most important and straightforward parameters to evaluate the efficiency of different substrates for mushroom production. The highest yield and BE were recorded from treatments containing wheat straw, which are also manifested in the growth parameters. Substrates containing water hyacinth gave intermediate yield and BE between wheat and *teff* straw, which was also observed for the growth parameters. Based on the results, water hyacinth biomass supplemented with 25% wheat straw can be considered an alternative substrate for oyster mushroom cultivation, provided that other vital parameters, such as nutritional content, are investigated for stronger justification. The least amount of yield and lowest BE was observed in substrates containing a higher proportion of *teff* straw. As mentioned earlier, the substrates with a lower C/N ratio such as *teff* straw provide the fastest pinhead formation, but the lowest in their mushroom yield [44]. Similarly, in the present study, the mushroom yield was lowest on *teff* straw even though this substrate provided the fastest pinhead formation. Overall, it can be justified that water hyacinth biomass can be good alternative substrate for oyster mushroom cultivation compared to *teff* straw.

### 4.4. Evaluation of the Economic Return on Different Substrate Combinations

Several parameters have to be considered to determine the economic return of oyster mushroom cultivation in different substrates. However, for a simple comparison purpose between the treatments, only the yield, the local price of oyster mushroom, and the costs of the substrates used here were considered. Based on this, using water hyacinth biomass had a high economic return compared to other substrates. This will have additional advantage as utilizing this biomass is also a means of controlling its negative effect on the aquatic ecosystem which is especially threatening huge lakes in Ethiopia such as Lake Tana. It should be noted that both types of straw (wheat and *teff*) are in demand in Ethiopia as they are utilized for cattle feed [45]. In addition, these types of straw are extensively used for reinforcing of mud or clay for house construction.

## 5. Conclusion

The results of this study demonstrate that water hyacinth biomass (an aquatic weed) alone or supplemented with wheat or *teff* straw can provide promising performances in oyster mushroom development, growth, yield, and biological efficiency compared to the competitive substrates (wheat and *teff* straw). In addition, based on most parameters, water hyacinth biomass is found to be better substrate for oyster mushroom cultivation compared to *teff* straw. This is a green light in using water hyacinth as an alternative substrate for mushroom cultivation, which will have a double advantage—cost reduction and controlling of the weed. The intrinsic factors such as nutrient, moisture-holding capacity, and other influencing variables in the different substrates need to be investigated further.

## Figures and Tables

**Table 1:** Description of the different treatments (substrate composition) used in this study; each treatment being replicated four times.

Treatments	Substrates composition by weight
T1	100% wheat straw (control)
T2	100% *teff* straw (control)
T3	100% water hyacinth
T4	75% wheat straw+25% water hyacinth
T5	50% wheat straw+50% water hyacinth
T6	25% wheat straw+75% water hyacinth
T7	75% *teff* straw+25% water hyacinth
T8	50% *teff* straw+50% water hyacinth
T9	25% *teff* straw+75% water hyacinth

**Table 2:** Effects of different substrate compositions on the days elapsed for mycelium invasion (colonization) (M1), pinhead formation (PF), and the first harvest (FH) of oyster mushroom after opening of the bags, 2021 Bahir Dar, Ethiopia.

Treatments	MI (M ± SD)	PF (M ± SD)	FH (M ± SD)
T1 (100% wheat straw)	21.50 ± 0.58^a^	5.25 ± 0.96^a^	9.25 ± 0.96^a^
T2 (100% *teff* straw)	24.25 ± 0.96^ab^	3.50 ± 0.58^b^	7.50 ± 0.58^b^
T3 (100% water hyacinth)	31.25 ± 0.96^c^	6.25 ± 0.96^a^	10.25 ± 0.96^ac^
T4 (75% wheat straw+25% water hyacinth)	24.00 ± 0.82^ab^	5.50 ± 0.58^a^	9.50 ± 0.58^acd^
T5 (50% wheat straw+50% water hyacinth)	25.00 ± 0.82^bd^	5.75 ± 0.50^a^	9.75 ± 0.50^ace^
T6 (25% wheat straw+75% water hyacinth)	27.75 ± 0.50^dec^	6.00 ± 0.00^a^	10.00 ± 0.00^acf^
T7 (75% *teff* straw+25% water hyacinth)	28.00 ± 0.82^ec^	3.75 ± 0.50^ab^	7.75 ± 0.50^ab^
T8 (50% *teff* straw+50% water hyacinth)	29.25 ± 0.50^c^	4.25 ± 0.96^ab^	8.25 ± 0.96^abde^
T9 (25% *teff* straw+75% water hyacinth)	29.75 ± 0.50^c^	4.50 ± 0.58^ab^	8.50 ± 0.58^abdef^

Means with different letters in the same column are significantly different (*p* ≤ 0.05). M = mean; SD=standard deviation.

**Table 3:** Effects of substrate composition on cap diameter (CD) and stalk length (SL) of oyster mushroom (*Pleurotus ostreatus*) growth, 2021, Bahir Dar, Ethiopia; *n* = 20.

Treatments	Mean of cap diameter (cm) ± standard deviation			
	1^st^ harvest	2^nd^ harvest	3^rd^ harvest	Total average∗
T1	7.5 ± 1.17^a^	7.3 ± 1.23^a^	6.6 ± 0.70^a^	7.14 ± 1.07^a^
T2	2.2 ± 0.69^b^	1.7 ± 0.54^b^	1.1 ± 0.04^b^	1.65 ± 0.67^bc^
T3	3.4 ± 0.51^bc^	3.3 ± 0.59^bc^	2.6 ± 0.29^c^	3.10 ± 0.57^c^
T4	6.6 ± 1.28^ade^	6.5 ± 0.92^ad^	5.8 ± 0.09^a^	6.30 ± 0.91^a^
T5	5.1 ± 1.75^cd^	5.1 ± 0.69^ace^	4.4 ± ±0.34^d^	4.84 ± 1.06^d^
T6	4.6 ± 0.93^cef^	4.3 ± 1.38^cdf^	3.6 ± 0.29^d^	4.18 ± 0.98^d^
T7	2.3 ± 0.59^bf^	2.2 ± 0.43^bf^	1.5 ± 0.28^be^	1.96 ± 0.55^bc^
T8	2.3 ± 0.36^bf^	2.4 ± 0.51^bf^	1.7 ± 0.27^bf^	2.14 ± 0.49^bc^
T9	2.7 ± 0.65^bf^	2.8 ± 0.35^bef^	2.1 ± 0.47^cef^	2.54 ± 0.56^bc^

Mean of stalk length (cm) ± standard deviation; *n* = 4				
T1	4.2 ± 1.45^a^	3.8 ± 0.51^a^	3.0 ± 0.26^a^	3.69 ± 1.22^ad^
T2	1.7 ± 0.86^b^	1.2 ± 0.22^b^	1.0 ± 0.02^b^	1.29 ± 0.54^bcf^
T3	2.5 ± 0.46^ab^	2.6 ± 0.32^ab^	1.6 ± 0.09^bc^	2.24 ± 0.53^bcdef^
T4	3.6 ± 0.96^ab^	3.8 ± 1.43^ab^	2.7 ± 0.30^ad^	3.41 ± 1.06^ad^
T5	3.2 ± 1.09^ab^	3.0 ± 1.37^ab^	2.2 ± 0.46^cd^	2.80 ± 1.05^acde^
T6	2.8 ± 0.41^ab^	2.5 ± 0.30^ab^	1.7 ± 0.25^c^	2.36 ± 0.57^cdef^
T7	1.9 ± 0.33^ab^	1.9 ± 0.43^ab^	1.1 ± 0.09^bc^	1.63 ± 0.48^bcef^
T8	2.1 ± 0.79^ab^	2.2 ± 0.81^ab^	1.4 ± 0.37^bc^	1.90 ± 0.72^bcdef^
T9	2.3 ± 0.95^ab^	2.3 ± 0.77^ab^	1.5 ± 0.36^bc^	2.03 ± 0.78^bcdef^

Means with different letters in the same column are significantly different (*p* ≤ 0.05) by the Tukey multiple range test.

**Table 4:** Effects of substrate composition on yield and biological efficiency of oyster mushroom (*Pleurotus ostreatus*) growth, 2021, Bahir Dar, Ethiopia; *n* = 20.

Treatment	Mean of weight (yield) in grams ± standard deviation			
	1^st^ harvest	2^nd^ harvest	3^rd^ harvest	Total yield (g)
T1	399.81 ± 9.52^a^	294.07 ± 9.63^a^	263.96 ± 8.44^a^	957.84 ± 25.14^a^
T2	130.04 ± 2.24^b^	125.08 ± 12.09^b^	94.97 ± 15.23^b^	350.09 ± 38.11^b^
T3	220.08 ± 9.24^c^	152.24 ± 9.15^c^	122.13 ± 3.27^b^	494.45 ± 20.11^c^
T4	320.17 ± 8.67^d^	270.24 ± 9.12^ad^	240.13 ± 9.12^ac^	830.54 ± 26.87^d^
T5	290.01 ± 12.45^d^	247.12 ± 12.50^d^	217.01 ± 0.83^cd^	754.14 ± 33.90^e^
T6	260.08 ± 1.08^c^	215.18 ± 10.95^e^	185.07 ± 0.95^d^	660.33 ± 32.97^f^
T7	179.06 ± 2.83^e^	134.52 ± 5.86^bc^	104.41 ± 5.86^b^	417.99 ± 4.06^bg^
T8	185.14 ± 5.07^e^	142.07 ± 8.16^bc^	111.96 ± 1.76^b^	439.17 ± 13.04^cg^
T9	203.80 ± 7.85^ce^	145.05 ± 9.02^bc^	114.94 ± 1.83^b^	463.79 ± 3.46^cg^

Mean of biological efficiency (%) ± standard deviation				
T1	39.98 ± 0.95^a^	29.41 ± 0.96^a^	26.40 ± 0.84^a^	95.78 ± 0.51^a^
T2	13.00 ± 1.23^b^	12.51 ± 1.21^b^	9.50 ± 1.53^b^	35.01 ± 0.81^b^
T3	22.01 ± 0.92^c^	15.22 ± 0.91^c^	12.21 ± 0.33^b^	49.45 ± 0.01^c^
T4	32.02 ± 0.87^d^	27.02 ± 0.91^ad^	24.01 ± 0.91^ac^	83.05 ± 0.69^d^
T5	29.00 ± 1.25^d^	24.71 ± 1.24^d^	21.70 ± 1.08^cd^	75.41 ± 0.75^de^
T6	26.01 ± 1.11^c^	21.52 ± 1.10^e^	18.51 ± 1.10^d^	66.03 ± 0.30^e^
T7	17.91 ± 1.28^e^	13.45 ± 0.59^bc^	10.44 ± 1.59^b^	41.80 ± 4.46^bc^
T8	18.51 ± 0.507^e^	14.21 ± 0.82^bc^	11.20 ± 0.18^b^	43.92 ± 1.30^bc^
T9	20.38 ± 0.78^ce^	14.51 ± 0.90^bc^	11.50 ± 0.18^b^	46.38 ± 1.34^bc^

Means with different letters in the same column are significantly different (*p* ≤ 0.05) by the Tukey multiple range test.

**Table 5:** Benefit-cost ratio of the different treatments (substrate composition) used in this study for oyster mushroom cultivation, 2021, Bahir Dar, Ethiopia.

Treatments	Total weight of mushroom harvested (g)	Total price (in ETB) of the mushroom harvested (B)	Cost (in ETB) of substrate per kg (C)	B:C
T1	957.84	287.352	13.33	21.56
T2	350.09	105.027	6.66	15.77
T3	494.45	148.335	1.00∗	148.34
T4	830.54	249.162	10.24	24.33
T5	754.14	226.242	7.16	31.60
T6	660.33	198.099	4.08	48.55
T7	417.99	125.397	5.24	23.93
T8	439.17	131.751	3.83	34.40
T9	463.79	139.137	2.41	57.73

The price for 1 kg mushroom during the study period was Birr 300 at the local market, and the costs of wheat and *teff* straws were Birr 13.33 and 6.66, respectively. B: C = benefit-cost ratio: ETB = Ethiopian Birr (currency): ∗Estimated labor cost to collect water hyacinth from the field (the lake).

## Data Availability

All data supporting the findings of this study are included in the paper; however, detailed data can be obtained from the corresponding author on request.
